# Optical and Electromechanical Design and Implementation of an Advanced Multispectral Device to Capture Material Appearance

**DOI:** 10.3390/jimaging10030055

**Published:** 2024-02-23

**Authors:** Majid Ansari-Asl, Markus Barbieri, Gaël Obein, Jon Yngve Hardeberg

**Affiliations:** 1 Department of Computer Science, Norwegian University of Science and Technology, 2815 Gjøvik, Norway; jon.hardeberg@ntnu.no; 2 Barbieri Electronic snc, 39042 Bressanone, Italy; markus.barbieri@barbierielectronic.com; 3 Laboratoire National de Métrologie et d’Essais, Conservatoire National des Arts et Métiers, 93210 La Plaine St Denis, France; gael.obein@lecnam.net

**Keywords:** appearance measurement, BRDF, spatially varying BRDF, BTF, multispectral imaging

## Abstract

The application of materials with changing visual properties with lighting and observation directions has found broad utility across diverse industries, from architecture and fashion to automotive and film production. The expanding array of applications and appearance reproduction requirements emphasizes the critical role of material appearance measurement and surface characterization. Such measurements offer twofold benefits in soft proofing and product quality control, reducing errors and material waste while providing objective quality assessment. Some image-based setups have been proposed to capture the appearance of material surfaces with spatial variations in visual properties in terms of Spatially Varying Bidirectional Reflectance Distribution Functions (SVBRDF) and Bidirectional Texture Functions (BTF). However, comprehensive exploration of optical design concerning spectral channels and per-pixel incident-reflection direction calculations, along with measurement validation, remains an unexplored domain within these systems. Therefore, we developed a novel advanced multispectral image-based device designed to measure SVBRDF and BTF, addressing these gaps in the existing literature. Central to this device is a novel rotation table as sample holder and passive multispectral imaging. In this paper, we present our compact multispectral image-based appearance measurement device, detailing its design, assembly, and optical considerations. Preliminary measurements showcase the device’s potential in capturing angular and spectral data, promising valuable insights into material appearance properties.

## 1. Introduction

A wide range of industries are currently producing and using materials that exhibit variations in their visual appearance under different lighting conditions and viewing angles. These industries span from architecture and textiles to fashion, packaging, lifestyle products, automotive, and film production. For example, in architecture, such materials find extensive use in enhancing interior designs, creating healthier and more aesthetically pleasing environments for occupants. Likewise, the application of these materials in exterior home decoration and facades contributes to urban beautification and the overall value of modern buildings for fostering inclusive, safe, resilient, and sustainable cities.

Moreover, the integration of such materials into everyday applications has led to a growing demand for producing products with diverse visual appearances using additive manufacturing techniques like 2.5D and 3D printing. Consequently, leading players in the printing industry are focusing on providing printers capable of producing samples with varying visual characteristics. Their efforts encompass hardware design, the development of software technologies, and the preparation of suitable printing materials.

The expanding use of complex materials across various sectors, coupled with the demand for accurate appearance reproduction, underscores the significant importance of material appearance measurement and surface characterization. In material reproduction, appearance measurement offers two key advantages: facilitating soft proofing and ensuring product quality control. Soft proofing is crucial as it ensures that designs align with intended specifications before physical production commences. By simulating the final output through soft proofing, errors can be identified and rectified in the early stages, reducing costly mistakes and minimizing material wastage. In this context, appearance measurement provides designers with insights into changes during the reproduction process, enabling necessary adjustments before production begins. Quality control, on the other hand, is conventionally conducted through visual inspection, which is subjective and susceptible to significant human errors. Therefore, employing appearance measurement as an objective evaluation method ensures dependable quality control.

Various methods have been proposed to capture visual appearance properties. Notably, the Bidirectional Reflectance Distribution Function (BRDF), initially introduced by Judd [1] and Nicodemus [2], offers a comprehensive model for describing the optical behavior of material surfaces. Essentially, BRDF characterize the reflectance of a material in all directions, which is particularly useful for describing gonioapparent materials whose color and visual attributes vary with illumination and viewing angles. While BRDF can be spectral in certain cases, it is not inherently so. However, spectral material reflectance refers to the property of a material that indicates how much incident light is reflected across different wavelengths. By converting reflectance data into colorimetric values, such as color coordinates in various color spaces like RGB, CIE XYZ, CIE Lab, etc., we can accurately portray the color of the material in a manner that is perceptually meaningful to humans. Another advantage of spectral reflectance is its independence from the device used, allowing for precise calculation of the material’s colorimetric information under any arbitrary illumination.

However, BRDF assumes uniform reflective properties across the entire object surface, which may not hold true in the presence of variations in texture or color. SVBRDF and BTF, as introduced by Dana et al. [3], can account for these variations. Both SVBRDF and BTF models are essentially functions of seven dimensions. However, differences in their underlying assumptions limit their applicability. Specifically, SVBRDF assumes that surfaces adhere to principles of energy conservation and reciprocity, restricting its use to flat and opaque surfaces. In contrast, the BTF model can describe surfaces with self-occlusion, self-shadowing, inter-reflection, and subsurface scattering. The seven dimensions common to these models include (x,y) coordinates representing the location on the surface, $\left(\theta_{i},\phi_{i}\right)$ and $\left(\theta_{r},\phi_{r}\right)$ spherical coordinates representing the incident and reflected directions, and the wavelength of light which is denoted as λ. The SVBRDF and BTF formulations are presented in the following equations [4]:

(1) $$\begin{array}{c} Y^{SVBRDF}=SVBRDF\left(x,y,\theta_{i},\phi_{i},\theta_{r},\phi_{r},\lambda \right),\\ Y^{BTF}=BTF\left(x,y,\theta_{i},\phi_{i},\theta_{r},\phi_{r},\lambda \right). \end{array}$$

The substantial dimensionality associated with measuring SVBRDF and BTF necessitates intensive data capture and analysis, making it a challenging task. Consequently, the design and construction of such measurement systems have always posed significant challenges. Nevertheless, numerous setups have been proposed, primarily within research circles. These setups can be categorized into several groups, with goniospectrophotometers and camera-light arrays being the most prominent.

Goniospectrophotometers typically involve a sample holder, often a robotic arm, that rotates the sample in all directions. Meanwhile, either a detector or a light source is rotated around the sample, while the other component remains fixed. Schwartz et al. [5] extensively reviewed their gonioreflectometer, developed and refined at the University of Bonn, aiming to conduct spatially varying and bi-directional measurements of the appearance of flat samples. In their setup, a robotic arm is employed to hold and rotate the sample, with a camera rotating on a semicircular rail system around the sample, while the light source remains fixed. While the original setup used a conventional red, green, and blue (RGB) camera, it was later replaced by a hyperspectral imaging system comprising a charge-coupled device (CCD) sensor and a tunable liquid crystal filter. Other configurations also fall under this category, such as setups where the sample remains fixed, while both the light source and camera are distributed across a hemisphere above the sample using two robotic arms [6]. Another approach, proposed by Azeri et al. [7], involves holding and rotating the sample along three axes using a rotation stage, moving a Light-Emitting Diode (LED) light source on a wheel around the sample, and maintaining a fixed RGB conventional camera to capture BTF data.

On the other hand, camera-light arrays aim to minimize mechanical movements to avoid vibrations and streamline data acquisition through parallel image captures. These setups typically consist of multiple cameras and light sources mounted on arcs or hemispheres above the sample. To achieve higher angular resolution, a rotation stage can be integrated into the setup. Havran et al. [8,9] proposed a miniaturized system for measuring spatially varying surface reflectance, represented by SVBRDF and BTF models, for onsite applications. Their design features a 25 cm diameter hemispherical gantry with 134 LED illumination modules distributed over it. Additionally, six cameras were installed on a stepper motor-driven arc with movements along the meridians. The entire gantry can be rotated about the sample’s surface normal, providing enhanced angular resolution. Schwartz et al. [5] extensively reviewed two camera-light arrays known as Dome 1 and Dome 2, developed at the University of Bonn. Dome 1 was designed to eliminate mechanical movements entirely, accommodating 151 compact cameras, with the built-in flash lamps of these cameras serving as light sources, ensuring identical camera and illumination directions. Conversely, Dome 2 consists of a fixed light dome featuring 198 LED lamps and a quarter-circle camera arc with 11 mounted cameras. It also incorporates a turntable sample holder to achieve different azimuthal angles.

While there exist various laboratory-based single-spot measurement setups that could potentially integrate a camera and corresponding algorithms for capturing BTF and SVBRDF, such as the Pab Gonio-Photometer PG2 [10], to the best of our knowledge only a limited number of compact image-based devices are commercially accessible for measuring SVBRDF and BTF. Among these, Chaos vrscans solely offers physical material samples measurement [11], whereas SIGHTTEX Q by SightTex s.r.o. [12] and the Total Appearance Capture material scanner (TAC7) by X-Rite Incorporated [13] both offer measurement services along with their respective devices. These systems quantify the reflectance of samples represented by SVBRDF and BTF models. TAC7 utilizes multispectral acquisition through a simplified active multispectral imaging technique, employing monochrome cameras and eight spectral light sources distributed across three filter wheels that each have ten bands.

Despite the occasional use of multispectral imaging techniques in a few SVBRDF and BTF acquisition systems [14,15], there remains a dearth of comprehensive investigations into such systems, both in terms of design and implementation, within the existing literature. Consequently, we have developed a novel compact multispectral image-based device for measuring material appearance. A concise introduction to this device was initially presented in [16]. This device is built around a new rotation table sample holder and passive multispectral imaging.

In our device, we have opted for a fixed camera while allowing the sample holder and illumination to rotate. This design choice offers several advantages. Firstly, a fixed camera reduces image registration errors compared to a moving camera, ensuring more accurate data collection. Additionally, it serves as a stable reference point for adjusting other components. Moreover, moving parts are at higher risk of collision, with multispectral cameras being particularly susceptible due to their high cost. Another technical advantage of a fixed camera is its stability during data transfer, minimizing the risk of interruption and data loss caused by cable movement.

Our device, inspired by goniospectrophotometers, features a simple rotation table as the sample holder, constructed using basic electromechanical components. This design replaces more expensive and less compact robotic arms typically found in such setups. A key innovation of our device is its compact integration of all components, unlike many laboratory setups where components are loosely arranged, making calibration challenging. Our goal was to create a device with fewer components, reducing size and cost while maintaining high precision.

By adopting passive multispectral imaging, our device enables multispectral measurements across all imaging geometries, an aspect often overlooked in the existing literature. This approach broadens the scope of multispectral imaging applications, offering new opportunities for research and analysis. In this paper, we delve into the challenges encountered during the optical and electromechanical design and assembly of this system. Therefore, our contributions in this paper can be summarized as follows:

- We present a new compact SVBRDF and BTF measurement device, centered around a rotation table and multispectral imaging.
- We address optical design considerations with regard to multispectral imaging, marking the first instance of such an investigation in the literature.
- We detail the hardware development process, covering design to implementation. The challenges encountered are thoroughly discussed.

This paper is structured as follows: Section 2 delves into the design of the entire system, encompassing mechanical design, rotation mechanism and calculations, the control system, and optical design, as well as camera and light selections. Section 3 provides insights into device assembly and alignment processes. Section 4 presents preliminary measurements, while Section 5 serves as the conclusion of this paper.

## 2. Design of the Device

### 2.1. Mechanical Design

The mechanical design and precise alignment of mechanical components hold immense significance in this device, particularly because it is designed to capture data that will be validated by metrological measurements. Metrological data acquisition demands exceptionally high accuracy. Therefore, the alignment and control of mechanical parts, which dictate both the device’s position and directional accuracy, must be meticulously managed. Furthermore, this instrument relies fundamentally on determining the positions of numerous points across a 3D surface, all of which involve rotations around multiple axes. Consequently, the mechanical design aspect warrants substantial attention.

**Figure 1:**
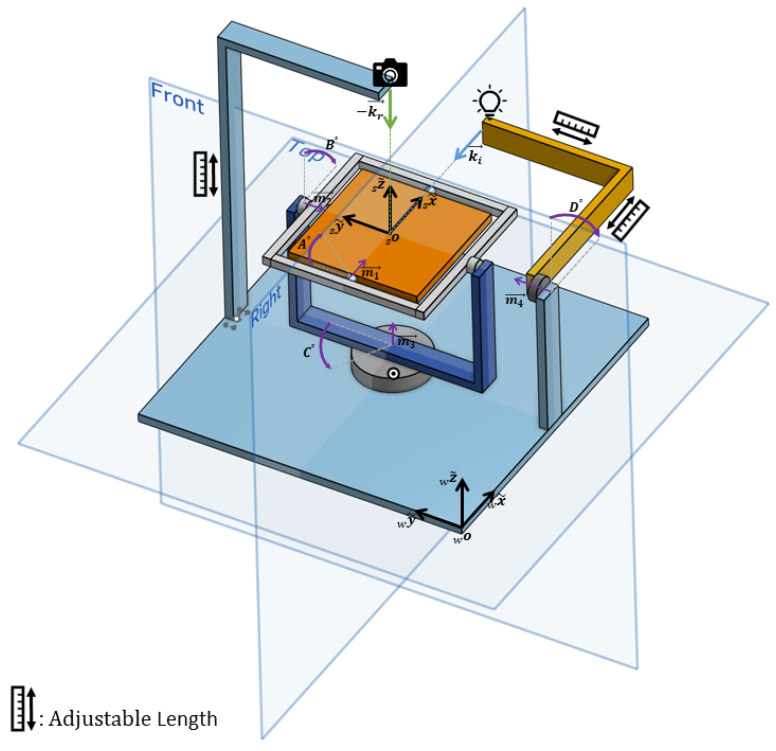
The overall conceptual mechanical design of the device, including a displayed sample and the world coordinate systems, is depicted. The axes of the stepper motors, along with their associated rotational directions, are represented in purple. The incident and inverse direction of reflection are provided in blue and green, respectively.

As illustrated in Figure 1, the mechanical components can be broadly categorized into three main parts: a fixed camera holder arm, an arm responsible for rotating the light source, and a rotational table serving as the sample holder. These three components, along with the electronic board, are integrated onto a base platform that can be securely affixed to an optical bench using four screws. Fundamentally, all physical parts and rotations are meticulously aligned with respect to a central point in space known as the “zero point”. The zero point essentially marks the intersection of three primary perpendicular planes: two of these are denoted as the “Front” and “Right” planes in Figure 1, while the third plane is parallel to the “Top” plane.

The central mechanical component is the turntable, responsible for holding the sample and executing rotations around three axes. It positions the sample in such a way that the central point over the sample aligns precisely with the zero point. Three stepper motors drive the rotation of the table, ensuring that all motor shaft axes intersect at the zero point while maintaining the zero point’s spatial position during rotations. The table’s configuration involves the outer motor, the third motor (M3), fixed on the base platform, rotating a half frame. The second motor (M2) is positioned at one end of this half frame, which in turn holds and rotates a full rectangular frame. The table itself is held inside this full frame and is rotated by the first motor (M1).

However, due to variations in sample thickness, the central point of the sample may not necessarily align with the zero point. To accommodate this, a mechanism has been implemented to adjust the height of the table. The table is not directly connected to the motor shaft; rather, two adjustable parts enable the table’s height to be modified using screws along the first motor’s axis. It is worth noting that the rotation of each of the motors M1-3 is transferred to the frames through a two-gear mechanism, which not only changes the direction of rotation but also shifts the shafts’ axes. These issues have been taken into consideration in the mechanical design. A cylindrical plastic cover is also fitted around the third motor to prevent cables from becoming entangled in the motor shaft.

The second crucial mechanical component is the light source arm, which provides the fourth degree of freedom (DF) for the device. The illumination source needs to rotate along a circular path around the zero point in the "Right" plane depicted in Figure 1. This arm comprises a segment fixed to the base framework, providing adequate height for the fourth motor’s axis to intersect with the zero point. The second L-shaped segment of the arm is adjustable in length on both sides to ensure precise positioning of the light source, accurately directed at the zero point. In the physical implementation of the instrument, a counterweight is employed to offset the arm’s weight, maintaining system balance and smooth operation.

The third mechanical component is the L-shaped camera holder arm, affixed to the base framework from the bottom. Similar to the light source arm, both sides of this arm are adjustable in length to position the camera at the intersection line of the “Right” and “Front” planes. This arrangement provides a variable camera-to-sample distance, ranging from approximately 30 to 45 cm. The camera is attached to the arm using a MENGS^®^ LP-64 precision leveling base tripod head, allowing for precise adjustment of the camera’s optical axis in all directions as well as the length of the arm in the L part through three screws.

We have implemented a comprehensive approach to address potential collisions within the system, incorporating both hardware and software safeguards. The system has been meticulously designed to mitigate the likelihood of collisions. To achieve this, specific rotation ranges and predefined rotation directions have been implemented for each motor, establishing clear operational boundaries. These parameters are enforced through the software, ensuring controlled and purposeful movements within designated ranges for optimal safety and performance. While the system offers flexibility in capturing incident-reflection combinations, it prohibits angles that could lead to collisions, thus ensuring a safety margin.

Nonetheless, the system may basically encounter two potential types of physical collisions: one occurs when the light source approaches and makes contact with the camera, and the other occurs when the light source comes into contact with the base platform. Therefore, the following measures have been implemented:

1. Camera-Light Source Collision: To prevent collisions between the camera and the light source, the software restricts incident-reflection combination directions that would require a light source arm motor angle of less than 5 degrees. This establishes a safety buffer around the camera and the light source, mitigating the risk of physical contact.
2. Light Source-Base Platform Collision: Similar to the first case, input angles are restricted by the software to avoid light source arm angles exceeding a predefined maximum. This precautionary measure prevents collisions between the light source and the base platform, enhancing the overall safety of the system.
3. TinyG Electronic Board Software Limits: Software limits have been activated within the TinyG electronic board, ensuring that the corresponding motors do not rotate beyond their predefined maximum limits. This additional layer of protection adds redundancy to the safety mechanisms, further reducing the likelihood of collisions.
4. Roller Lever Switches: As an extra safety measure, roller lever switches have been installed to halt the machine in the rare event that both software limits and the TinyG electronic board limits fail to prevent unwanted motor rotations.

By combining these hardware and software safety features, we have significantly minimized the risk of collisions within the system, providing a robust and secure operating environment for our device.

### 2.2. Rotation Mechanism

To encompass all possible combinations of hemispherical incident and reflection directions, a total of four DFs are required. In this specific setup, three of these DFs are allocated to a motorized turntable, allowing it to rotate along the axes of three stepper motors. The fourth DF is assigned to an arm responsible for rotating the light source. In this arrangement, the camera remains fixed on the camera holder, and the convergence point of the camera’s optical axis and the light source, along with the axes of all four motors, resides at the center of the table surface, known as the “zero point”. The table surface’s height can be adjusted to accommodate samples of varying thickness, ensuring that the sample’s central point consistently aligns with the device’s zero point.

Within this configuration, three distinct coordinate systems can be considered. The first one is fixed on the device’s base and remains stationary as the motors rotate. This coordinate system, also referred to as the world or lab coordinate system, is defined by unit basis vectors {x~w,y~w,z~w}, where the *w* prescript denotes the world. It constitutes a Euclidean space because all three axes are mutually orthogonal. Since the axes of this coordinate system remain unchanged during motor rotations, any rotation about these axes is classified as extrinsic rotation.

The second coordinate system is the sample coordinate system, defined by unit basis vectors {x~s,y~s,z~s}. Also known as the moving space, its origin is set at the device’s zero point. Similarly, it is a Euclidean space in which incident and reflection vectors are defined. An important characteristic of this space is its co-rotation with the motor rotations, i.e., the frame moves with motor rotations with respect to the world coordinate system. Therefore, any rotation along the axes of this space is referred to as intrinsic rotation.

The third coordinate system, of particular importance in defining the device’s features, is constructed from the rotation axes of the three stepper motors that manipulate the table, namely M1, M2, and M3. This coordinate system is known as the motors coordinate system, with its axes denoted as $\tilde{m_{1}}$, $\tilde{m_{2}}$, and $\tilde{m_{3}}$, corresponding to the rotation axes of motors M1, M2, and M3, respectively. This coordinate system holds significance because converting input incident-reflection hemispherical directions from the sample coordinate system is required to be into rotations around the axes of this space. Unlike the previous spaces, this one is not Euclidean because $\tilde{m_{1}}$ is not always perpendicular to $\tilde{m_{3}}$. Consequently, sample rotations in all directions achieved through motor rotations may not always be of the same type, whether intrinsic or extrinsic. However, an initial position is defined for the entire device, wherein all three coordinate systems coincide, and absolute rotations are measured from that position. In this specific configuration, motor rotations in the order of $\tilde{m_{1}}$, $\tilde{m_{2}}$, and $\tilde{m_{3}}$ provide extrinsic rotations about x~w, y~w, and z~w, respectively.

### 2.3. Spherical to Motors Coordinate Conversion

The hemispherical incident-reflections within the sample coordinate system are provided to the device as input, either by the user or through the inner hemisphere scanning algorithm. As a prerequisite to transmitting rotation commands to the machine, these directions must undergo conversion into stepper motor rotation angles, namely *A*, *B*, *C*, and *D*, corresponding to motors M1, M2, M3, and M4, respectively. While angle transformation mathematics and equations for rotation stage sample holders [17,18] as well as robotic arm sample holders [19,20], have been presented in some references, it is important to note that these equations are typically specific to the configuration of the setup. As a result, they may need to be derived individually for each setup based on its particular configuration. In this section, building upon the previously detailed rotation mechanism in Section 2.2, we will derive the conversion equations that translate hemispherical incident-reflection directions into motor rotation angles.

**Figure 2:**
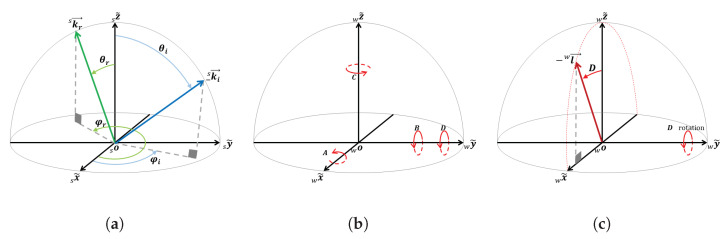
Sample and world coordinate systems with motor rotations: (**a**) sample coordinate system and incident-reflection directions with their spherical coordinates; (**b**) world coordinate system with motor rotations directions in the initial position; (**c**) world coordinate system with the light source vector.

In Figure 2a, the sample coordinate system is depicted, and the unit vectors for incident kwi→ and reflection kwr→ are illustrated for arbitrary incident and reflection directions in spherical coordinates, denoted as $\left(1,\theta_{i},\phi_{i}\right)$ and $\left(1,\theta_{r},\phi_{r}\right)$, respectively, where θ represents the zenith angle and ϕ denotes the azimuthal angle. Assuming that the origins of all three coordinate frames (world, sample, and motors) are placed at the device’s zero point, and their corresponding axes coincide in the initial position, stepper motors M1, M2, and M3 can induce rotations equivalent to extrinsic rotations around x~w, y~w, and z~w, as shown in Figure 2b. It is important to note that, for simplicity, all incident, reflection, camera, and illumination directions are treated as vectors with their starting points at the origin of the coordinate systems, avoiding the need for negative signs.

In this setup configuration, the angles *A*, *B*, and *C* can be considered as a set of Euler angles, which are three sequentially applied elemental rotations about the axes of the world coordinate system: x~w, y~w, and z~w, respectively. These elemental rotations are always sufficient to reach any target frame or vector. Hence, we employ extrinsic rotations represented by a set of Tait–Bryan angles in the x~w**-**y~w**-**z~w order. This entails starting with a rotation of *A* degrees about x~w, followed by a rotation of *B* degrees around y~w, and concluding with a rotation of *C* degrees about z~w.

Consequently, within this device, any point or vector denoted as Pw in the world space can be reached from a vector in the sample space Ps through a rotation from the sample coordinate to the world coordinate Rsw. This rotation is composed of three elemental rotations: R(xw,A), a rotation equal to *A* degrees about x~w; R(yw,B), a rotation equal to *B* degrees about y~w; and R(zw,C), a rotation equal to *C* degrees about z~w.

Equation (2) formulates the matrix equation for the overall rotation and its corresponding elemental rotations. The matrices for elemental rotations and the overall rotation are presented in Equation (3) and Equation (4), respectively:

(2) Pw=RswPs=R(z~w,C)R(y~w,B)R(x~w,A)Ps;

(3) R(x~w,A)=1000cosA−sinA0sinAcosA,R(y~w,B)=cosB0sinB010−sinB0cosB,R(z~w,C)=cosC−sinC0sinCcosC0001;

(4) Rsw=cosCcosBcosCsinBsinA−sinCcosAcosCsinBcosA+sinCsinAsinCcosBsinCsinBsinA+cosCcosAsinCsinBcosA−cosCsinA−sinBcosBsinAcosBcosA.

A square matrix, denoted as *R*, qualifies as a rotation matrix if and only if $R^{-1}=R^{T}$, i.e., its inverse is equal to its transpose, and the determinant of *R* is 1 (det(R)=1). Therefore, based on the rotation from the sample coordinate to the world coordinate as outlined in Equation (4), the rotation from the world coordinate to the sample coordinate can be represented as Rws=RswT, and in matrix form it can be expressed as follows:

(5) Rws=cosCcosBsinCcosB−sinBcosCsinBsinA−sinCcosAsinCsinBsinA+cosCcosAcosBsinAcosCsinBcosA+sinCsinAsinCsinBcosA−cosCsinAcosBcosA.

Upon the computation of rotation matrices that map the sample and world coordinates to each other, rotation angles can be determined as functions of the input incident-reflection spherical coordinates through vector analysis.

As depicted in Figure 2a, the unit vectors for the incident and reflection directions can be expressed in the spherical coordinate system as follows:

(6) ksi→=sinθicosϕisinθisinϕicosθi,ksr→=sinθrcosϕrsinθrsinϕrcosθr,

where ksr→ and ksi→ represent the reflection and incident directions in the sample frame. Conversely, because the camera remains fixed on the device, its direction is consistently oriented along the z~w axis. Following rotations, the reflection direction aligns with the camera direction. Thus,

(7) ksr→=Rwsz~w.

By substituting Equations (5) and (6), as well as z~w=001 into Equation (7), we obtain:

(8) sinθrcosϕrsinθrsinϕrcosθr=Rws001=−sinBcosBsinAcosBcosA,

upon dividing the second row by the third row, the value of *A* is derived as:

(9) $$A=atan2(\sin\phi_{r}\sin\theta_{r},\cos\theta_{r}),$$

where

(10) $$atan2\left(x,y\right)=\left\{\begin{array}{c} \tan^{-1}\left(\frac{y}{x}\right)\;if\;x>0\\ \tan^{-1}\left(\frac{y}{x}\right)+\pi \;if\;x<0\\ \frac{\pi }{2}\;if\;x=0\;and\;y>0\\ -\frac{\pi }{2}\;if\;x=0\;and\;y<0 \end{array},\right.$$

and the first row of Equation (8) yields the value of *B* as follows:

(11) $$B=\sin^{-1}\left(-\cos\phi_{r}\sin\theta_{r}\right).$$

As depicted in Figure 2c, the light source is rotated by motor M4 within the x~w−z~w plane. Therefore, the unit vector representing the light source in the world coordinate system can be expressed as:

(12) lw→=sinD0cosD.

Similarly, following the rotations, the incident direction aligns with the light source direction. Therefore, in the world coordinate system,

(13) lw→=Rswksi→,

and by substituting Equations (6) and (13) into Equation (13), we obtain:

(14) sinD0cosD=Rswsinθicosϕisinθisinϕicosθi.

To simplify the calculations and eliminate the need for *D* in the calculations, we substitute Equation (4) into this equation and focus on the second row. Ultimately, *C* is calculated as follows:

(15) $$\begin{array}{c} C=atan2(\sin\theta_{r}\sin\phi_{r}\cos\theta_{i}-\sin\theta_{i}\sin\phi_{i}\cos\theta_{r},\\ \cos\theta_{r}\left(\sin\theta_{i}\cos\phi_{i}\cos\theta_{r}-\sin\theta_{r}\cos\phi_{r}\cos\theta_{i}\right)+\sin\theta_{i}\sin\phi_{r}\sin^{2}\theta_{r}\sin\left(\phi_{r}-\phi_{i}\right)). \end{array}$$

As illustrated in Figure 2c, both the fixed camera direction, which aligns with z~w, and the light source lie within the x~w−z~w plane. Furthermore, following all rotations, the camera direction and light source coincide with the reflection and incident directions, respectively. Consequently, the incident and reflection vectors lie in the same plane, and their inner product is utilized to determine the value of *D*.

(16) cosD=ksi→·ksr→,

and by substituting the incident and reflection vectors with their polar coordinates, as shown in Equation (6), and performing the inner product, we obtain the value of *D* as follows:

(17) $$\cos D=\sin\theta_{i}\cos\phi_{i}\sin\theta_{r}\cos\phi_{r}+\sin\theta_{i}\sin\phi_{i}\sin\theta_{r}\cos\phi_{r}+\cos\theta_{i}\cos\theta_{r}$$

(18) $$D=\cos^{-1}\left(\sin\theta_{r}\sin\theta_{i}\cos\left(\phi_{r}-\phi_{i}\right)+\cos\theta_{r}\cos\theta_{i}\right).$$

With the conversion equations in place, our system can effectively explore the entire spectrum of hemispherical directions. The motorized rotations are carefully constrained to ensure comprehensive coverage. Specifically, motor M1 and M2 operate within the range of −90 to 90 degrees (*A* and *B* angles), while Motor M3 has a rotation span of −180 to 180 degrees (*C* angle). Lastly, motor M4, controlling the light source, sweeps from 0 to 180 degrees (*D* angle). These prescribed ranges allow our setup to meticulously navigate and capture in incident and reflection angles across the entire hemispherical domain.

#### Coordinate Conversion Evaluation

To assess the accuracy of the coordinate conversion equations through simulation, we employed a back-projection strategy. A defined set of incident-reflection direction pairs was utilized as the input for the transformations, yielding motor rotation angles. This process involved calculating motor rotations for 62,500 incident-reflection combinations. The distribution of cameras and illuminations spanned across the intersection of meridians, varying from −180 to 180 azimuth angles with a 15-degree interval, and parallels from 0 to 90 zenith angles with a 10-degree increment.

**Figure 3:**
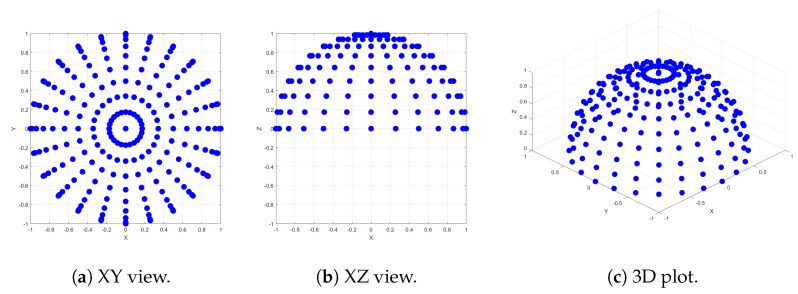
Spherical positions representing the test set of imaging geometries. The identical set was utilized for both illumination and camera positions.

The arrangement of illumination and camera positions across the hemisphere, distributed along meridians and parallels with specific azimuth and zenith angles, has been previously employed in other studies, such as [8]. Given the flexibility of our system, capable of capturing data in various geometries, we selected an arbitrary test set of 62,500 incident-reflection directions, ensuring coverage across the entire hemisphere with reasonable increments. It is important to note that this set may not directly correspond to the geometries utilized in our data capture process. Both illumination and camera positions were chosen identically. Additionally, we intentionally included some overlapping positions at azimuthal angles of +180 and −180, as well as at zenith angle 0, to assess the accuracy of our conversion equations. The positions for both incident and reflected directions are illustrated in Figure 3.

Subsequently, we back-projected these motor rotation angles to obtain incident-reflection coordinates. This was achieved by considering the camera direction along the z~w axis and computing the light source in the world coordinate using Equation (12). These vectors were then back-projected to the sample coordinate space using the rotation matrix from Equation (5) derived from the motor rotation angles. Finally, we converted the directions from the Cartesian coordinate system to spherical coordinates.

The evaluation involved calculating the differences between the input incident-reflection directions and their corresponding back-projected directions. The results demonstrated a predominantly one-to-one transformation behavior for the 62,500 combinations. The back-projected directions precisely matched their corresponding input directions, except in cases where either the incident or reflection direction had θ equal to 0, aligning with the surface normal. Specifically, when $\theta_{r}=0$, $\phi_{r}$ was consistently back-projected to zero ($\phi_{r}=0$). In cases where $\theta_{i}=0$, $\phi_{i}$ was typically back-projected to random values unequal to their corresponding input values. Indeed, these particular cases do not compromise the accuracy of the transformations. When θ=0, it signifies that the vector is directed towards the apex of the hemisphere, where θ alone is sufficient to describe its orientation, rendering ϕ irrelevant. Consequently, we can confidently assert that the directions are effectively back-projected to match their respective input directions. Overall, this analysis reinforces our conclusion that the coordinate conversion equations function robustly and accurately.

### 2.4. Electronics and Control Units

**Figure 4:**
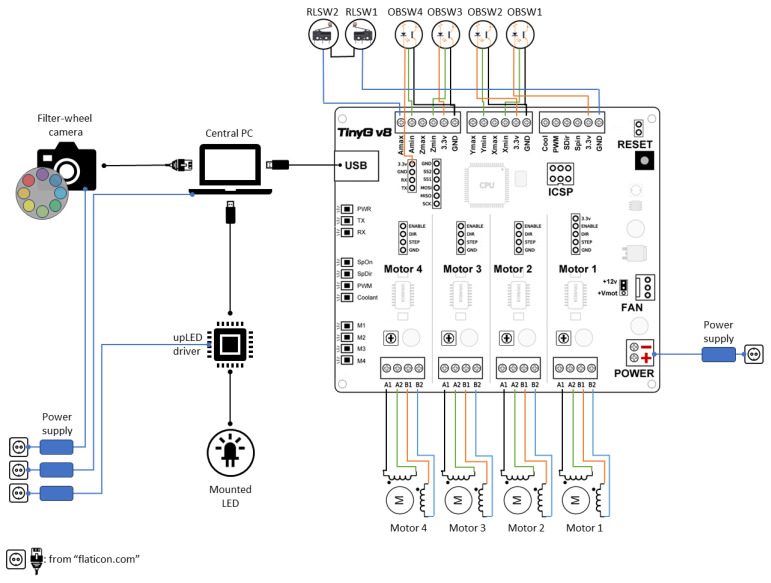
The schematic of control units and electronic components. The central component of this system is a PC, responsible for coordinating communication, synchronization, and control among all elements. The sample holder table and light source arm are regulated by the TinyG v8 multi-axis motion board. To bring the device to the zero position, four optical barrier switches (OBSW 1–4 ) are employed, while two roller lever microswitches (RLSW 1–2) prevent damage to the light source arm. The light source operates autonomously through the upLED LED driver. TinyG board layout from [21].

The overarching conceptual framework of the control system and electronics is comprehensively depicted in Figure 4. At the core of this system is a personal computer (PC), which orchestrates the communication, synchronization, and control of all components. A dedicated software, developed as part of this project, serves as the nerve center, processing data and enabling seamless interaction with the hardware. Central to the control of the sample holder table and light source arm is the TinyG v8 multi-axis motion control system [21]. This specialized board, tailored for precision motion control applications like CNC machining, plays a pivotal role. It boasts an Atmel ATxmega192A3U - 8-bit AVR microcontroller and four TI DRV8818 stepper motor drivers, rendering it ideal for our purpose of controlling four motors with a single board. According to the manufacturer’s specifications, the drivers on the TinyG are rated for 2.5 A per winding per motor, but they can handle up to 3 amps with proper cooling, and they provide microstepping capabilities of up to 8 microsteps. The board interfaces with the PC via Universal Serial Bus (USB), and its microcontroller locally interprets G-codes.

Motors M1, M2, and M3 belong to the TRINAMIC SY42STH38-1206B type, featuring an input voltage of 4 V and a nominal phase current of 1.2 A. The fourth motor is a TRINAMIC QSH6018-86-28-310, equipped with an input voltage of 4.17 V and a nominal phase current of 2.8 A. We have configured each motor to move in steps of 1.8 degrees, defining a complete 360-degree revolution. Specifically, for motors 1 to 3, which drive translational axes with their respective motor shafts connected to the table through gears, the gear ratio is set at 4:1. Consequently, we have adjusted the travel per revolution to 90 mm for these axes, resulting in an overall 360-degree travel per revolution.

In Figure 4, four optical barrier switches, OBSW 1–4, are prominently featured and serve as essential components for the homing procedure, also known as zeroing. This procedure establishes the device’s absolute coordinates by positioning it at a known zero location. The absolute device coordinate system serves as the reference for calculating motor rotations. During the homing procedure, motors initiate movement in predefined directions until they make contact with their corresponding optical switches. The motors operate sequentially in the order M3, M1, M2, and M4. The microprocessor detects the homing of a motor when the optical switches, configured in the normally closed mode, emit a falling edge transition signal.

To ensure a swift and complete shutdown of the machine in emergency situations, thereby preventing potential damage, a reset button and machine kill switches have been implemented. When the reset button on the TinyG board is pressed, it halts the control system, bringing the machine to a stop and depowering the motors. However, it is important to note that the motors remain depowered only as long as the reset button is kept pressed. The light source arm, responsible for rotating the illumination on a circular path around the sample, is constrained within specific angles through soft switches configured on the microcontroller. For added safety, two roller lever microswitches, RLSW 1–2, are positioned on the top and both sides of the arm. These switches are wired in series in the normally closed mode, ensuring that the machine is immediately deactivated if any of the switches are pressed due to contact with an object.

The TinyG board is connected to a power supply with an output voltage and current rating of 12 V and 3 A, respectively. The light source operates independently and is controlled by a dedicated USB LED driver known as upLED [22]. The installed LED is connected to the upLED driver through an M8 × 1 connector, while the driver itself is linked to and controlled by the main PC via a USB2 connection. The camera communicates with the PC by receiving commands and transmitting images through the Gig-E interface standard. It is powered separately through a dedicated power supply.

### 2.5. Multispectral Camera Selection

We had access to three multispectral cameras employing two different technologies: Silios CMS-C and Silios CMS-S, equipped with Spectral Filter Array (SFA), and Pixelteq SpectroCam, a multispectral filter wheel camera. Selecting the optimal camera involves assessing various factors, including size, weight, resolution, pixel pitch, spectral responsivity, dynamic range, and digital output bit depth. This section provides detailed specifications for each camera and outlines the rationale for our selection.

Silios CMS-C [23] is an SFA-based multispectral camera featuring a Bayer-like mosaic filter placed in front of a commercial complementary metal-oxide semiconductor (CMOS) sensor. The sensor boasts an original resolution of 1280 × 1024 pixels, but due to its 3 × 3 Bayer-like filter configuration, the resulting spectral image is reduced to a 426 × 339 macropixel resolution. This camera captures eight narrow-band channels and one panchromatic band. The narrow-band filters span the spectral range from 430 to 700 nm, with Gaussian-like filter transmission functions exhibiting an average full width at half maximum (FWHM) of 40 nm bandwidth and centered at 440 nm, 473 nm, 511 nm, 549 nm, 585 nm, 623 nm, 665 nm, and 703 nm. The panchromatic band remains relatively consistent across the entire visible wavelength range. The sensor features a pixel pitch of 5.3 μm, and its digital output has a 10-bit depth, communicated via either USB 3.0 or Gig-E interface standards. The camera’s dimensions are approximately 62 × 62 × 31 mm, with a maximum weight of 110 g.

**Figure 5:**
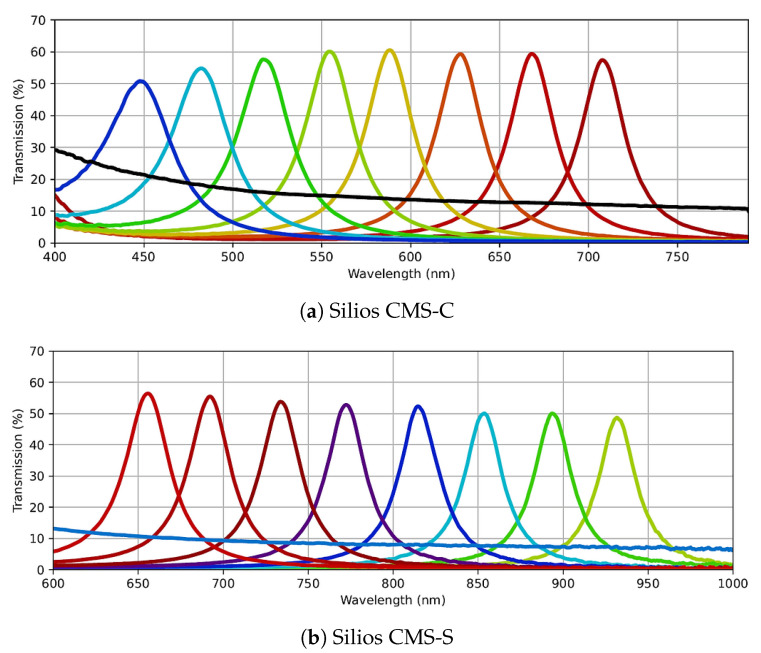
The spectral filter transmissions of SFA cameras: (**a**) the narrow-band filters of Silios CMS-C span the spectral range from 430 to 700 nm, with an average FWHM of 40 nm bandwidth. (**b**) Silios CMS-S filters cover the spectral range from 650 to 930 nm. Images from [23].

Silios CMS-S [23] shares the same specifications as Silios CMS-C, except for the spectral range and filter transmissions. Silios CMS-S predominantly covers the spectral range from 650 to 930 nm, extending beyond the visible range. Figure 5 illustrates the spectral filter transmissions for both Silios CMS-C and Silios CMS-S cameras.

**Figure 6:**
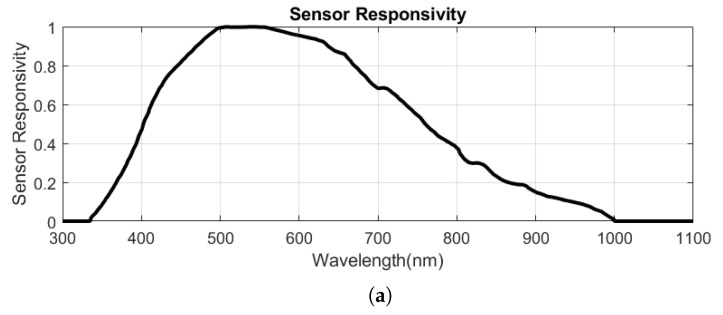

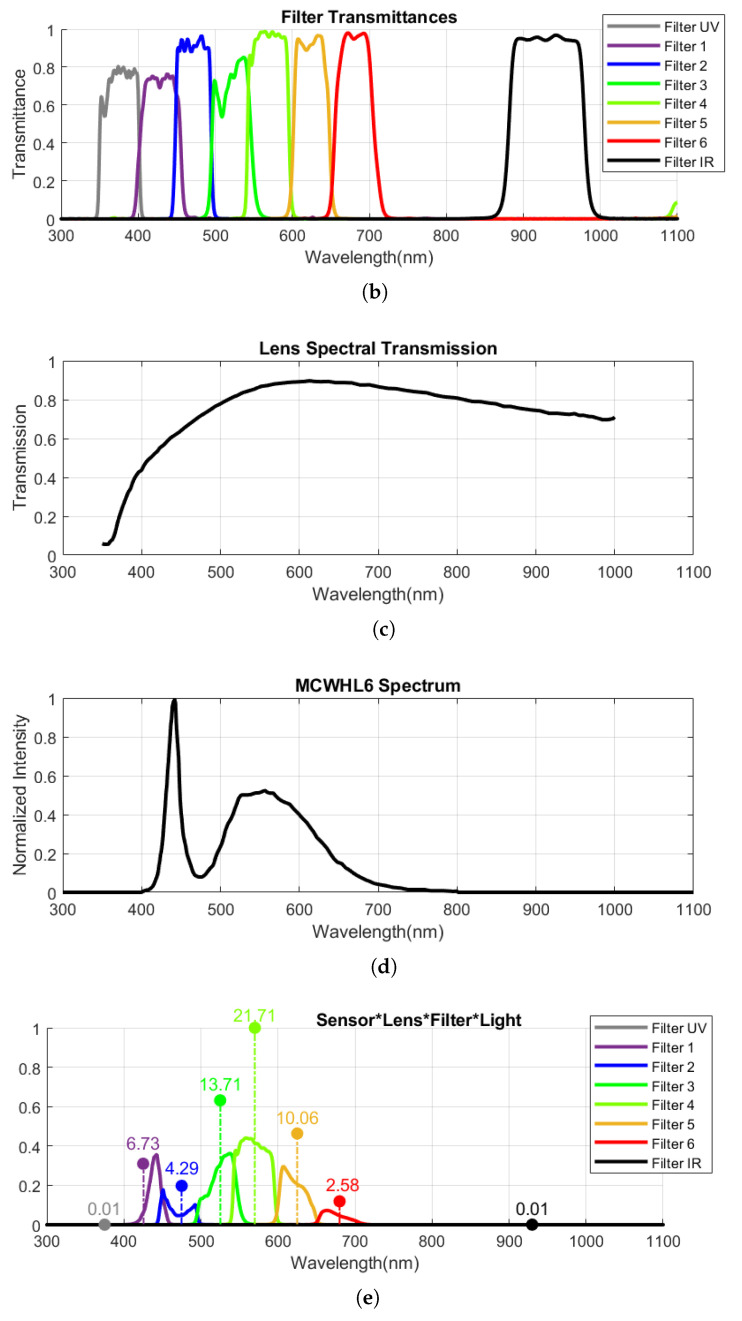
The spectral specification of the SpectroCam filter wheel camera and its integration with the Thorlabs MCWHL6 mounted LED: (**a**) the normalized sensor responsivity, data provided by the manufacturer; (**b**) the normalized transmittance of the filters. All eight filters are exhibited; however, Filter UV and Filter IR were omitted from the experiment due to inadequate light intensity. Data provided by the manufacturer; (**c**) the spectral transmission of the Carl Zeiss Distagon 2.8/25 ZF-IR lens. Data from [26]; (**d**) the normalized MCWHL6 mounted LED spectrum; (**e**) the product of the Sensor, filters, and light spectra. Additionally, the stem plot displays the normalized integral values of spectral channels, with numerical annotations representing the actual integral values. The stem bars are positioned over the respective central wavelengths for each channel.

The SpectroCam (VIS + NIR) multispectral filter wheel camera [24] employs a CCD silicon panochromatic sensor with spectral responsivity spanning from 350 nm to 1050 nm. It incorporates a filter wheel housing eight interchangeable filters in front of the sensor. Commercially, there are at least 145 interference filters with various spectral characteristics available for this camera [25]. The spectral filter transmittances initially installed on the camera are depicted in Figure 6. The filter central wavelengths are 375 nm, 425 nm, 475 nm, 525 nm, 570 nm, 625 nm, 680 nm, and 930 nm. The first seven filters have a bandwidth of about 50 nm each, while the last filter has a bandwidth of approximately 100 nm. The camera produces images with a resolution of 2456 × 2058 pixels and a pixel pitch of 3.45 μm. Its digital output can reach up to 12 bits, and communication occurs through a Gig-E interface standard. The camera’s physical dimensions are 136 × 124 × 105 mm, with a weight of 680 g.

Table 1 summarizes the key specifications of all three cameras. In comparison to the SpectroCam, the Silios CMS-C and Silios CMS-S cameras are notably lightweight and suitable for applications where the camera must be held by a robotic arm and moved within space. Their compact dimensions also allow more room for positioning the light source above the sample and avoids occlusion. SFA cameras offer a significant advantage with their snapshot property, enabling the capture of all spectral channels in a single shot. In contrast, filter wheel cameras require multiple shots, one for each spectral channel, while the filter wheel rotates and positions a new filter in front of the sensor. Additionally, mechanical rotation of the filter wheel can introduce misalignment in images of different spectral channels if the camera holder is not adequately secured. Despite the advantages of SFA cameras, we opted for the SpectroCam in our device. Its broader spectral sensor responsivity and filter interchangeability enhance its flexibility, particularly for spectral reflectance reconstruction purposes. With its high resolution, increased digital output bit depth, and smaller pixel pitch, the SpectroCam proves significantly more suitable for SVBRDF and BTF measurements, where spatial resolution and precision are critical. Another advantage of filter wheel technology is the ability to construct customized cameras tailored to specific requirements, such as the number of channels, filter types, and dimensions, allowing for adaptability to future instrument designs.

However, due to the spectral limitations of the light source in the UV and IR regions, only six filters within the visible range are utilized (as depicted in Figure 6). The sensor’s original low dynamic range, covering only 2.5 orders of magnitude, falls short for various materials. Consequently, a multispectral high dynamic range (HDR) imaging technique employing bracketing is employed [27].

### 2.6. Lens Selection and Optical Design

In optical design, numerous parameters must be considered, including the F-number, lens focal length, pixel size, sensor dimensions, sample distance, depth of field (DoF), spatial resolution, and field of view. These parameters are interrelated, and their relationships are pivotal in optical system design. Various optical design approaches have been proposed in the literature, often involving simplifications to streamline the design process. Němcová et al. introduced a novel optical design strategy that commences from the ground up [28]. This strategy aligns well with the specific requirements of BTF and SVBRDF design, making it a proper choice for lens selection and other predefined parameters within our application.

**Figure 7:**
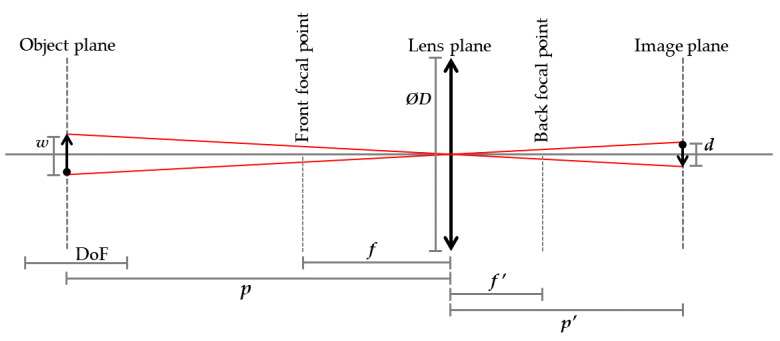
A simplified diagram illustrating thin lens optics following Cartesian convention to measure distances from the lens. Variables *f* and *p* are negative, representing the lens focal length and the object distance in front of the camera, respectively. Conversely, $f^{\prime }$ and $p^{\prime }$ are positive, denoting the lens focal length on the sensor side and the image distance. *d* is a negative value with its absolute representing the diameter of the Airy disk, while *w* is the diameter of the back-projection of the Airy disk onto the object. ∅D represents the aperture diameter, and DoF is depth of field.

A simplified geometry of thin lens optics with Cartesian notation featuring the optical parameters discussed in this paper is presented in Figure 7. Němcová et al. assumed a thin lens approximation and equated the circle of confusion (CoC) to the Airy Disk, resulting in an aberration-free optical design. Assuming a diffraction-limited system in paraxial space, they derived formulas for calculating various optical parameters. They demonstrated that, in such a system, combinations of three independent optical design input variables dictate all other optical and geometric parameters. Importantly, they revealed that, at a given wavelength, the maximum DoF, the distance between the minimum and maximum object distance for which the image is in focus, is solely determined by the object distance in front of the lens and the spatial resolution over the object. This relationship is expressed as follows:

(19) $$\mathrm{DoF}=\frac{2p^{2}Lw^{2}}{p^{2}L^{2}-w^{4}},$$

where depth of field, denoted as DoF, is determined by two parameters: the object distance, represented as *p*, and the spatial resolution, denoted as *w*. In this equation, *L* is a constant specific to the given wavelength λ, defined as L=2.44λ.

As a result, having knowledge of any two parameters among DoF, spatial resolution, and object distance, it becomes sufficient to determine only one parameter from the remaining optical factors in order to calculate all the optical design parameters. In our specific application, we employ a precise ChArUco target-based corner detection algorithm during the registration process, making it desirable for the entire ChArUco board to remain in focus during measurement. Therefore, we set the DoF to be equal to the size of the ChArUco board, which is 10 cm. Additionally, to minimize the risk of the camera being obstructed by the light source, we choose the object distance to be at its maximum by positioning the camera at the highest available height, resulting in p=46.5 cm. Furthermore, we employ a fixed focal length lens with $f^{\prime }=25$ mm, which serves as the third predefined parameter. One rationale for considering the measurement distance and focal length as input parameters is that the wavelength may influence the remaining variables to be calculated, leading to different values for various spectral channels. However, these two parameters cannot be altered during the measurement, whereas other parameters can be adjusted in the software. For instance, changes in pixel size may be managed using pixel binning.

Given these clarifications, our application aligns well with the design strategy outlined in [28], which assumes that DoF, object distance, and lens focal length are provided as inputs, and the maximum attainable resolution *w* is determined by the following relationship:

(20) $$w=\sqrt{\frac{-pL(p+\sqrt{p^{2}+{\mathrm{DoF}}^{2}})}{\mathrm{DoF}}}.$$

The entrance pupil diameter, denoted as ∅D, and consequently, the f-number represented as F/#, can be expressed as follows:

(21) $$\emptyset D=-L\frac{p}{w},$$

(22) $$F/\#=\frac{f^{\prime }}{\emptyset D}.$$

The corresponding Airy disk diameter, represented as *d*, and taking into account the “point concept” where only resolved points are considered, assuming that the pixel size is equal to the Airy disk’s radius, the pixel size can be calculated using the following equation:

(23) $$d=\frac{wf^{\prime }}{w+f^{\prime }}\;\mathrm{and}\;pix=\left|\frac{d}{2}\right|.$$

Utilizing this optical design strategy, we have calculated the optical parameters for different spectral channels of the filter wheel camera, and the results are presented in Table 2. Throughout these calculations, the measurement distance, lens focal length, and DoF have remained constant for all channels. As demonstrated in the table, the wavelength has a noticeable impact on the calculated variables. The spatial resolution ranges from 112 dots per inch (DPI) for filter 1 to 89 DPI for filter 6. Consequently, the pixel size varies from 6.43 to 8.10 μm, while the physical camera sensor has a pixel pitch of 3.45 μm. Importantly, the f-number is computed to range from 11.73 to 9.31, and the differences in values are insubstantial. We have chosen to utilize the Carl Zeiss Distagon 2.8/25 ZF-IR lens, which offers optical parameters within this range. Concerning the f-number, the closest value provided by the selected lens to the calculated values is approximately F/#=11, and this f-number has been chosen for our application.

### 2.7. Light Source

**Figure 8:**
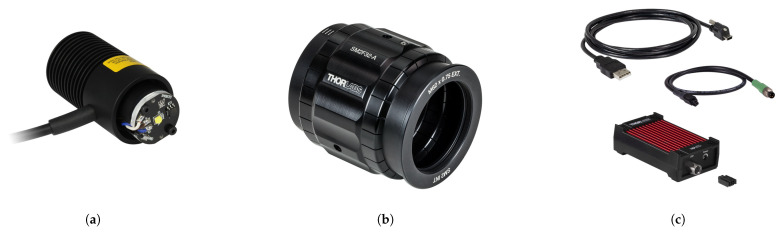
Different components of the light source: (**a**) the MCWHL6 LED, serving as the light source’s core, consists of a single white-spectrum LED soldered onto a PCB. The PCB includes an EEPROM chip for storing LED information and is attached to a heat sink; (**b**) Thorlabs SM2F32-A adjustable collimation adapter; (**c**) upLED™ constant current LED driver. Images from Thorlabs [31].

**Figure 9:**
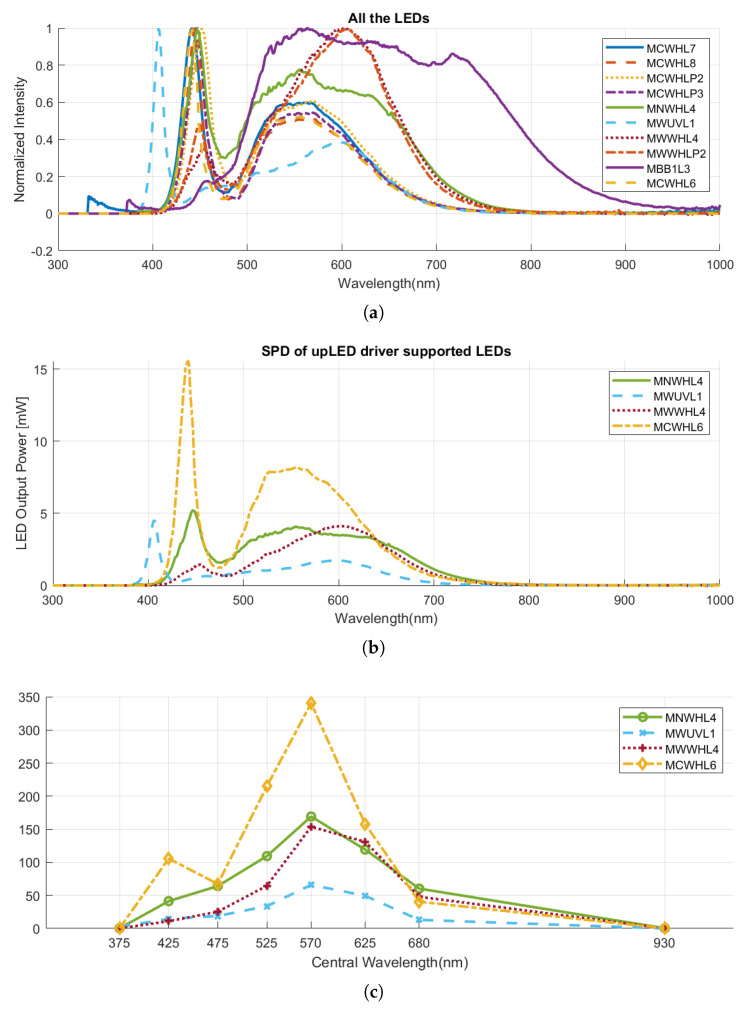
The spectral characteristics of LEDs to choose from: (**a**) the normalized SPDs of all the available Thorlabs white and broadband mounted LEDs. Data from Thorlabs [31]; (**b**) the SPDs of Thorlabs LEDs that are able to be controlled by upLED driver; (**c**) the integral of the multiplication of the LED SPD by the filter transmittances, lens transmission, and normalized spectral sensor responsivity.

The light source in our setup comprises various components that require careful consideration during the design and implementation phase. Figure 8 displays all these elements, encompassing:

1. LED: We have selected the Thorlabs MCWHL6 uncollimated mounted LED as the core of our light source. This LED emits cold white light with a correlated color temperature (CCT) of 6500 K and a typical output power of 1430 mW. It is soldered to a printed circuit board (PCB), which also features an electrically erasable programmable read-only memory (EEPROM) chip to store essential LED information, such as current limits, wavelength, and forward voltage [29]. The spectral power distribution (SPD) of the chosen LED is illustrated in Figure 9, and the procedure for selecting the LED is elucidated in the following section.
2. PCB: The PCB is responsible for holding the LED and providing basic electronics and wiring. It is affixed to the end of a heat sink to ensure proper thermal management.
3. Heat Sink: A heat sink is employed to dissipate the heat generated by the LED effectively, preventing overheating and ensuring the LED’s stable operation.
4. Adjustable Collimation Adapter: The MCWHL6 LED has a broad viewing angle of 120 degrees, causing the emitted light to diverge widely. To address this, we utilize a collimator. Collimating the light ensures that it reaches the sample with consistent direction at every point on the surface. This is crucial for capturing SVBRDF and BTF data, where collimated lighting is essential. Additionally, collimation concentrates the light onto the sample area, resulting in higher irradiance, which is particularly important for spectral imaging when each spectral channel is limited by a narrow filter. We have chosen the Thorlabs SM2F32-A adjustable collimation adapter [30]. This adapter has a 5 cm diameter output, anti-reflection (AR) coating in the 350–700 nm range, a lens with a focal length of 32 mm, and allows adjustment and lens positioning via a rotating ring with a travel range of 20 mm. The collimator is depicted in Figure 8b.
5. Constant Current LED Driver: To control the LED’s intensity, we utilize the upLED™ LED Driver, as illustrated in Figure 8c, which features a USB 2.0 interface [22]. This driver can provide a maximum LED current of 1.2 A and offers a 1 mA LED current setting resolution. It can be controlled manually via a potentiometer on the device or through dedicated software. Importantly, it provides an SDK for C++ programming environments, which is well-suited for our application. A power supply with a 12 V and 1 A output is used to power the driver and provide the necessary power for the LED.

By carefully selecting and integrating these components, we ensure that our light source meets the specific requirements of our application, providing both collimated uniform illumination and precise control over it.

#### 2.7.1. LED Selection

In the process of selecting LEDs, Thorlabs products were chosen due to their extensive range of LED types with varying spectral properties. Additionally, Thorlabs provides all the necessary components for an illumination unit, encompassing optical components to control units equipped with suitable programming SDKs. Our investigation focused on Thorlabs’ mounted LED products, specifically targeting white and broadband LEDs covering the visible range, as our goal was spectral reconstruction and precise color measurement. Thus, our exploration was confined to the following LED models: MCWHL7, MCWHL8, MCWHLP2, MCWHLP3, MNWHL4, MWUVL1, MWWHL4, MWWHLP2, MBB1L3, and MCWHL6. The normalized SPDs of all the available Thorlabs white and broadband mounted LEDs are illustrated in Figure 9a.

Given that our system aims to be an integrated device with comprehensive control capabilities through a dedicated software for synchronized data capture, the chosen light source must be controllable using an electronic driver and accessible through a C++ SDK. The Thorlabs white and broadband mounted LEDs are controlled by different drivers. To ensure seamless integration into our system, we required LEDs controlled by the upLED™ constant current LED driver, the only driver which supports C++ SDK. Consequently, our LED selection was narrowed to four options: MNWHL4, MWUVL1, MWWHL4, and MCWHL6. Table 3 presents a summary of the technical specifications for these LEDs, and their absolute SPDs are depicted in Figure 9b.

Considering Table 3, the MCWHL6 LED model stands out with better specifications among the available options. Notably, its higher output power and irradiance offer a significant advantage for our multispectral camera, especially given the limited bandwidth of its spectral bands, requiring increased energy for effective noise reduction during measurements.

For a more detailed examination of intensity within the range of each spectral band, we performed a calculation involving the multiplication of the LED SPD by the filter transmittances and normalized spectral sensor responsivity. Subsequently, we computed the integral within the corresponding range, resulting in relative values denoting the effective energy received by the sensor considering spectral sensor responsivity for each multispectral channel. This energy is then converted into pixel values through the opto-electronic transfer function (OETF) of the sensor. Higher values indicate an improved combination of the illumination and imaging system, leading to more precise capture of information within that channel. Additionally, these values provide insights applicable to setting channel-specific exposure times, mitigating over- and under-exposure concerns. Figure 9c illustrates the results, emphasizing the generally higher performance of MCWHL6 compared to the other LEDs.

#### 2.7.2. LED Evaluation through Spectral Reconstruction

The Color Rendering Index (CRI) [32] and Television Lighting Consistency Index (TLCI) [33] are commonly employed metrics for illuminations assessment. However, as highlighted in [8], CRI is primarily suitable for incandescent, fluorescent, and high-intensity discharge (HID) luminaires, but not for white LEDs. On the other hand, TLCI, specifically designed for LED quality assessment, relies on color differences of outputs of a full imaging pipeline simulation that involves a standardized television camera, which is essentially a conventional RGB camera. This does not align with our device utilizing a multispectral camera. Therefore, instead of using the aforementioned indices, we chose to evaluate the color and spectral performance of LEDs through a spectral reconstruction procedure. The four previously chosen LEDs undergo a spectral reconstruction evaluation process, and the ultimate selection of the LED is based on both its performance and the provided technical specifications.

Basically, the formulation of a multispectral image is similar to that of conventional tristimulus images, differing only in its increased number of channels. In a simplified noiseless form, the camera response for a given stimulus is expressed as follows:

(24) $$\rho_{n}=\int_{\lambda }s_{n}\left(\lambda \right)i\left(\lambda \right)r\left(\lambda \right)d\lambda ,$$

Here, $\rho_{n}$ represents the camera response in the $n^{th}$ channel. r(λ) and i(λ) denote the spectral reflectance of the object and the SPD of the light source, respectively. The variable λ stands for wavelength, and $s_{n}\left(\lambda \right)$ signifies the spectral sensor responsivity corresponding to the $n^{th}$ channel. In a multispectral camera with *N* channels, there are *N* sensor responsivities corresponding to channels, i.e., $s_{n}\left(\lambda \right)\in \left\{s_{1}\left(\lambda \right),s_{2}\left(\lambda \right),\ldots ,s_{N}\left(\lambda \right)\right\}$. Converting Equation (24) into matrix form results in

(25) ρ=SIR,

considering *N*, *P*, and *J* as the number of channels, number of samples, and number of wavelengths, respectively. ρ is a matrix of size N×P representing camera responses of *P* recorded samples, each containing *N* values corresponding to *N* camera sensors. Consequently, *S* is an N×J matrix, and *I* is a J×J diagonal matrix denoting the SPD of the illumination. The dimensions of *R* are also J×P, indicating it contains *P* samples and *J* wavelengths for each.

In such a formulation, reflectance recovery addresses the estimation of spectral reflectances, *R*, from multi-stimulus values, ρ. Considering a new matrix, *M*, as the multiplication of camera responsivities by illumination SPD as known parameters, M=SI, Equation (25) becomes:

(26) ρ=MR.

If *M* were known and invertible, the solution for reflectance recovery would be as straightforward as an inverse problem, where

(27) $$\hat{R}=M^{-1}\rho ,$$

and $\hat{R}$ is the estimation of spectral reflectance. However, *M* is not always invertible, and this solution does not yield sufficient results as it is highly sensitive to noise. Numerous works in the literature aim to address this estimation problem and find a matrix that transforms measured multispectral data into spectral reflectance space, denoted as *W* in the equation $\hat{R}=W\rho$. A widely used linear method is Wiener estimation [34], which by ignoring noise, suggests calculating *W* as follows:

(28) $$W=R_{t}R_{t}^{T}{\left(SI\right)}^{T}{\left(SIR_{t}R_{t}^{T}{\left(SI\right)}^{T}\right)}^{-1},$$

where $R_{t}R_{t}^{T}$ represents the autocorrelation matrix of the training spectra.

For the spectral reconstruction experiment, the Wiener estimation method is employed with a k-fold cross-validation technique, utilizing the Munsell dataset, which consists of 1269 color patches with known spectral reflectances. The purpose is to ensure that the same dataset serves as both the training and testing dataset while guaranteeing that the testing set has not been used in the training process. In this study, a fourfold cross-validation is applied, where the dataset is divided into four complementary subsets. In each round, one subset is designated as the testing set, and the remaining subsets constitute the training dataset. This process is repeated until all subsets have been utilized as the testing set. The choice of k = 4 in folding the entire dataset is considered reasonable, allowing 75 percent of the data for training and 25 percent for testing in each iteration.

The choice of the reconstruction error metric is another crucial aspect of the evaluation process. Error metrics are commonly divided into colorimetric and spectral categories, and we have incorporated both in our assessment. For colorimetric errors, the CIE DE94 color difference has been computed under three distinct standard illuminations: D65, A, and F2. To calculate DE94, the reconstructed spectrum and its corresponding ground truth are transformed into XYZ tristimulus values under a specific illuminant, utilizing two-degree standard observer color matching functions. Subsequently, CIE LAB values are derived and employed in the computation of CIE DE94 to quantify the colorimetric error. For spectral error metrics, we have utilized the root mean square error (RMSE) and the goodness of fit coefficient (GFC).

The experiment was replicated for the four LEDs, and the outcomes are detailed in Table 4. While the spectral error metrics exhibit close proximity for all LEDs, MCWHL6 demonstrates a marginally superior performance in terms of colorimetric evaluation. Consequently, taking into account its superior reconstruction performance alongside favorable technical specifications, such as a higher CCT and output power, we have ultimately opted to employ the MCWHL6 mounted LED as the designated light source in our device.

## 3. System Realization

This device is fundamentally based on multiple rotations around four stepper motors’ axes, enabling incident-reflection hemispherical direction coverage across a hemisphere over the sample. Consequently, the accurate placement of components, alignment of motions and different parts, as well as synchronization of rotations and control units become of paramount importance.

**Figure 10:**
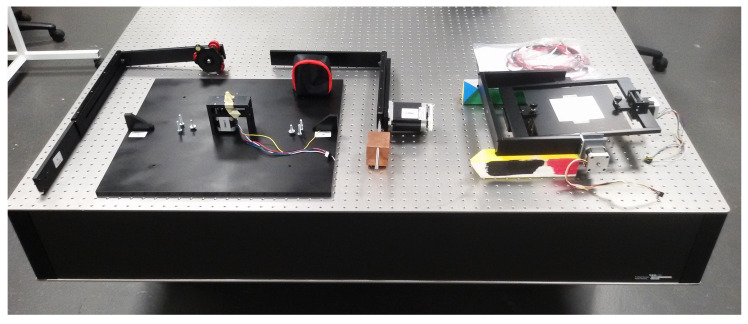
The primary mechanical components of the instrument arranged on the optical bench before assembly. From left to right in the figure, the components include the camera holder, the base platform with the third stepper motor mounted and arm screws, the light source rotational arm with the corresponding counterweight, and finally, the rotation table.

Figure 10 depicts the main mechanical elements of the instrument organized on the optical bench prior to assembly. On the left side, the initial component is the adjustable camera arm featuring a MENGS^®^ LP-64 precision leveling base tripod head to secure the multispectral camera. Initially, the tripod head was positioned with its screw facing downward, but it was later rotated 90 degrees to align with the optical axes of the camera, ensuring perpendicularity to its tripod socket. Following this is the base framework of the device, housing the central third stepper motor and screws for arm fixation. Adjacent is the light source arm, similarly adjustable in height, with its stepper motor pre-installed, and a counterweight employed for maintaining balance during rotation. The counterweight’s position on its shaft is determined based on the light source’s weight and the force introduced by the light source cable. Additionally, a sliding component was later incorporated into the arm to secure and adjust the length of the light source in the upper section. On the right side, the rotation table is situated, featuring the main table and full frame with coupled half frame holders precisely mounted, along with the first and second stepper motors.

**Figure 11:**
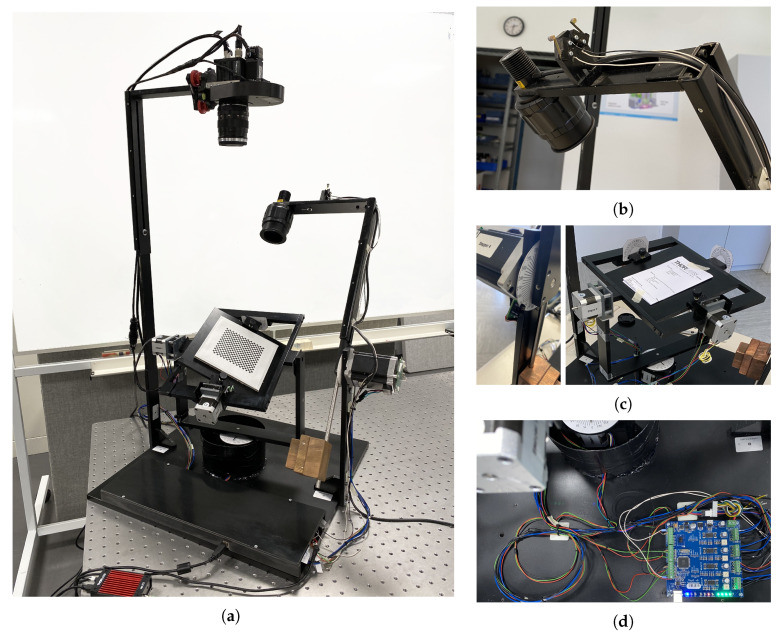
The final implementation phase: (**a**) the device on an optical bench in the laboratory; (**b**) the roller lever microswitches wired in series in the normally closed mode for additional device safety; (**c**) the four protractors mounted for visual inspection and alignment; (**d**) the TinyG control board and its wiring.

The rotation table is the first component meticulously mounted on the base framework. Subsequently, the camera and light source arms are affixed to the device body, followed by the installation of the multispectral camera and the light source. The counterweight for the light source arm is adjusted to match the light source’s weight, and both sides of its L-shaped structure are configured to align with the camera’s position and the zero point. The direction of its illumination is also fine-tuned using a screw on the arm. Similarly, the vertical section of the camera arm is set to its maximum height, and the length of the horizontal part, along with the camera’s optical axis, is aligned with the tripod head where the camera is mounted. Figure 11 provides a view of the fully assembled device during the final implementation phase while Figure 11b shows a close-up photo of the roller lever microswitches wired in series in the normally closed mode for additional device safety.

To achieve precise alignment of the camera with respect to the table, a software adjustment mode has been implemented. In this mode, the camera captures video, which is displayed in the software with a crosshair. The screws on the tripod head are then used to finely adjust the camera’s position with respect to the ChArUco board on the table. The crosshair in the camera video should align with the crosshair on the calibration board situated on the table.

Furthermore, four protractors shown in Figure 11c are mounted on the hardware surrounding the four rotation axes of the device to facilitate visual inspection and alignment. These protractors aid in defining the absolute rotation angles for each axis as well.

Finally, the control board is installed as depicted in Figure 11d and the necessary wiring is completed. To safeguard the electronic board, it is shielded with a plastic cover. Additionally, to prevent cables from becoming entangled in the motor shafts, a cylindrical plastic cover is placed over motor M4.

### 3.1. Adjustment Mechanisms and Accuracy

In order to ensure precise alignment of the various components in the device and to accommodate potential misalignments that may occur during hardware implementation or over time, we have incorporated several adjustment mechanisms into the instrument. Figure 1 and Figure 11 illustrate these mechanisms, which include adjustable L-shaped arms for both the camera and light source, allowing for vertical and horizontal adjustments via a sliding bar mechanism. Additionally, the camera holder tripod head enables angle adjustments for both the illumination and camera using screws on their respective slide bars and tripod head mounts.

Furthermore, we have mounted a ChArUco board on the rotational table for registration purposes. In its current configuration, designed for the measurement of nearly flat samples, manual adjustment of the sample position over the ChArUco board is facilitated through the use of double-sided adhesive films. Minor misalignments can then be resolved using the registration algorithm.

To assist with adjustments, the software features a live video mode with a crosshair overlay. Moreover, to address any biases that may arise in the hardware, an option to define motor offsets is available, allowing for correction of deviated motor positions, particularly during homing procedures.

To investigate the alignment of the camera with respect to the zero point, particularly during rotations, we conducted an experiment. In our device, the zero point is expected to align with the central pixel of the camera image and ideally should remain fixed at this location throughout rotations. However, due to imperfections in design and electromechanical implementations, deviations may occur, causing the zero point to appear in different locations within the image.

**Figure 12:**
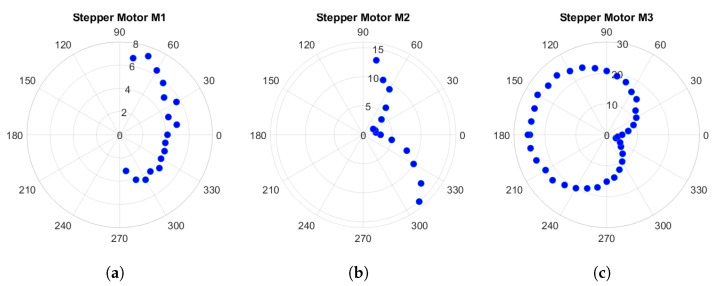
Deviation of the camera axis from the zero point during rotations of motors M1 to M3. Azimuth represents the rotation angle, with deviations depicted in pixels in the polar plots: (**a**) deviations in M1 rotation; (**b**) deviations in M2 rotation; (**c**) deviations in M3 rotation.

In this experiment, we individually rotated each motor within its operational range in 10-degree increments and captured images at each step. Subsequently, we calculated the deviation from the central pixel. Figure 12 illustrates these deviations in terms of pixels. The polar plots in the figure display the rotation angle (azimuth) and corresponding deviations in pixels. Due to technical constraints, we were unable to measure deviations at a few angles for motors M1 and M2.

As depicted in the figure, the maximum deviation occurs for motor M3 at a rotation angle of 180 degrees, amounting to 25 pixels. This deviation corresponds to approximately 1.6 mm in the sample space, which is comparable to the 1.3 mm observed in Lightdrum [8]. This deviation of 1.6 mm in the sample space corresponds to a maximum deviation of 0.2 degrees of the camera optical axis at an imaging distance of 45 cm in the device. To address this slight error, we employ image processing techniques.

### 3.2. Angular Repeatability

This apparatus is designed to precisely measure the appearance of materials from various angles, making angular repeatability essential in a goniometric device. Angular repeatability denotes the ability to return to a predetermined position consistently in successive attempts, ensuring that repeated measurements at the same angle produce consistent results. This level of precision is vital for reliable data collection and analysis. In our device, homing is integral to angular repeatability as it establishes the coordinates for absolute angular rotation of motors. To achieve this, we have implemented light barrier switches to position stepper motors accurately at absolute coordinate points. However, there may be concerns regarding the angular repeatability of this setup compared to motors equipped with absolute angular encoders, owing to potential hysteresis behavior in the light barrier switches.

To mitigate hysteresis during homing and enhance homing repeatability, we implemented a specific routine. Homing is performed only once and during the initialization stage. Upon completion of any measurement and subsequent shutdown of the machine, it returns to a predefined position and halts, ready for the next measurement. If its position has been altered for any reason, prior to restarting the machine, we manually adjust the table and illumination arm close to the starting positions. Subsequently, the motors are rotated in the opposite direction of their homing rotations by a certain degree to ensure they are within the desired quadrant and to prevent any switches from being triggered before homing. Since the homing rotation direction is pre-defined and homing occurs at a specified edge of the switches, the motors consistently halt at a particular position, effectively mitigating hysteresis in the switches and enhancing homing repeatability.

To assess the angular repeatability of the setup, we conducted an experiment involving repeated homing procedures. Following the completion of system adjustments, we executed homing ten times and analyzed the repeatability. Given the fixed position of the camera in our setup, it served as the reference for our investigation. Subsequent to each homing iteration, we captured an image and compared the data across images. The ChArUco board utilized for camera calibration and image registration features a 10 by 10 chessboard pattern with 50 markers sporting unique IDs within the white patches. Leveraging a corner detection algorithm, we extracted the corners of the ChArUco board and compared corresponding points across different homing images. Due to occlusion of three markers by additional drawings on the ChArUco board, each image yielded 47 identified markers, totaling 188 points per image as each marker gives four corners. This homing procedure was repeated ten times. We computed the distances between the corresponding points for each repetition and calculated the mean values. Subsequently, we determined the minimum, maximum, average, and standard deviation distances between corresponding points in each pair of images. The maximum distance observed across all points in all repetitions was 3.32 pixels. Since the distances were measured for an adequate number of points across the entire chart in each repetition, and the distances predominantly clustered around the average, we consider this a reliable measure and utilize it in our calculations, resulting in one distance per image. The maximum of these distances observed across all repetitions was 0.87 pixels, with a mean value of 0.75 pixels and a standard deviation of 0.13 pixels. The minimal distances observed between corresponding points in images indicate that the device consistently returns to the same position during successive homing attempts, demonstrating an acceptable level of precision and thereby enhancing the repeatability of the setup.

To demonstrate homing repeatability and deviations in terms of distances in the sample space and motor angular rotations, we utilized the known size of the ChArUco board and its projection in images and employed proportional and basic mathematical relationships. Initially, we determined the average and standard deviation of detected corners in images from 10 repetitions to assess repeatability. Subsequently, we computed the distance of each corner to the center of the image, representing the projection of the radius of the virtual sphere that the corner traverses during motor rotations in the world space. Given that homing involves the rotation of three stepper motors, pixel shifts cannot directly translate into angular rotations of motors. Thus, we made certain assumptions to calculate angular deviations in homing for stepper motor M3. As the motor M3 rotates the table around the camera optical axis and both the table and its image remain perpendicular to it during the rotation, pixel shifts in images can be straightforwardly converted into physical values in the sample space. To evaluate the repeatability of motor M3 in homing, we assumed the worst-case scenario where deviations are solely attributed to motor M3, disregarding contributions from M1 and M2. The dimensions of each chessboard square on the ChArUco board are 1 cm, projecting to 155 pixels in images at the device’s zero position, where the board is perpendicular to the camera optical axis. Consequently, corner distances from the zero point, along with standard deviations, can be translated into physical scales in the sample space. Utilizing these physical values, we calculated the actual angular rotation deviations for all 188 detected corners. Our findings reveal that the average real angular rotation errors range from 0 to 0.43 degrees, with an average and standard deviation of 0.1 and 0.07 degrees, respectively.

### 3.3. Limitations

Incorporating the practical limitations and considerations that were not accounted for during the design phase, this section addresses the limitations introduced by the device.

Upon deriving the spherical to motors coordinate conversion equations in Section 2.3, we realized a constraint regarding the movement of motor M4, responsible for light source rotation. In theory, while scanning all incident-reflection direction combinations over the hemispherical space, motor M4 is expected to operate within the [0, 180] degrees interval. However, due to the light source arm being set at its maximum length during practical implementation, the rotation is limited to a maximum of 120 degrees. Consequently, the device cannot access incident-reflection direction combinations where their corresponding D angle exceeds 120 degrees. To quantify the impact of this limitation, we calculated the motor rotations for 62,500 incident-reflection combinations, where cameras and illuminations are distributed along the intersection of meridians from −180 to 180 azimuth angles with a 15-degree step, and parallels from 0 to 90 zenith angles with a 10-degree step. Among these combinations, 3458 exhibited D angles beyond the 120-degree limit, constituting approximately 5 percent of the total combinations.

Another limitation arises from the size of the camera and light source, along with their rotations within a plane, potentially leading to collisions. The likelihood of a collision is contingent upon the lengths of the camera holder arm and the light source holder. Even in cases where they do not collide, there is a possibility of mutual occlusion. Consequently, we have set a minimum acceptable angle of 5 degrees to mitigate these issues. Given that the camera remains fixed at the 0 angle, D cannot be less than 5 degrees, effectively restricting the device from reaching approximately 1.5 percent of the aforementioned 62,500 incident-reflection combinations. Similar to the previous limitation, this does not imply that the camera and illumination cannot access angles less than 5 degrees relative to the surface normal.

## 4. Example Measurement

We conducted initial measurements following the assembly of the device to assess its functionality in capturing angular and spatial data. The recorded images are promptly transferred and stored on a PC via a Gig-E interface during the measurement process. Following the completion of the measurement, a post-processing stage is performed using a dedicated software program. This stage involves rectifying, registering, and cropping images using the ChArUco board markers positioned on the rotation table. A detailed description of the algorithms and an in-depth evaluation of the data capture are beyond the scope of this paper and constitute our planned future work. In this section, we present some preliminary measurements and discuss their outcomes.

**Figure 13:**
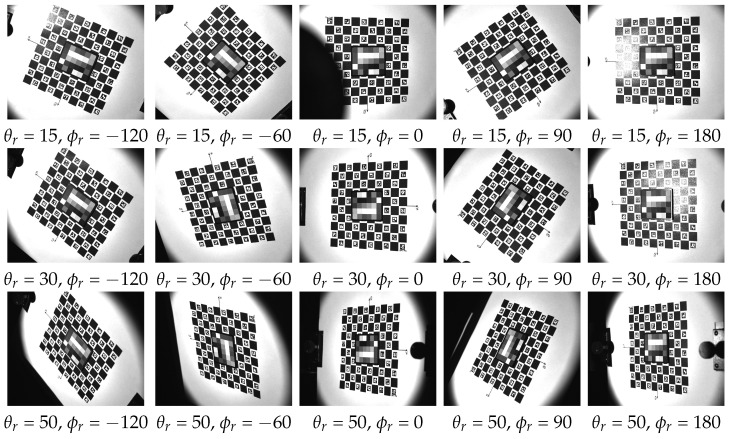
Captured images. The incident light is fixed at $\theta_{i}=20$ and $\phi_{i}=0$, while the reflection direction changes. The images are arranged in rows that correspond to different angles of reflection ($\theta_{r}=15$, 30, and 50 from top to bottom) and in columns that correspond to different directions of reflection ($\phi_{r}=-120$, −60, 0, 90, and 180 from left to right).

**Figure 14:**
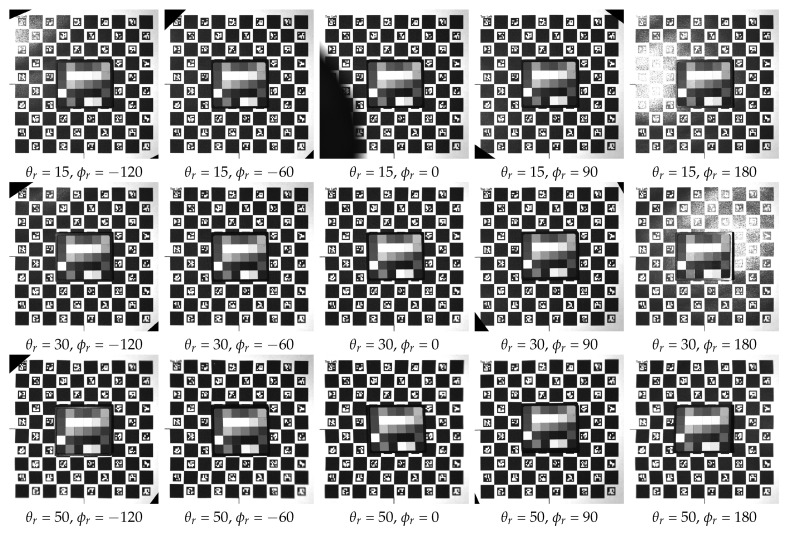
Registered images as output of post-processing stage. The imaging geometries are the same as in Figure 13.

In the first experiment, we maintained the incident direction at $\theta_{i}=20$ and $\phi_{i}=0$. We captured images of a ColorGauge Micro Target [35] at 15 reflection directions, encompassing directions with zenith angles of $\theta_{r}=15$, 30, and 50, and azimuth angles of $\phi_{r}=-120$, −60, 0, 90, and 180. Specifically, we focused on images from the fourth spectral channel centered at 575 nm, as illustrated in Figure 13. These images were then processed through post-processing stages, resulting in registered images displayed in Figure 14. As expected, the output images from post-processing were rectified and registered, creating a form of being captured under a perpendicular reflection direction.

**Figure 15:**
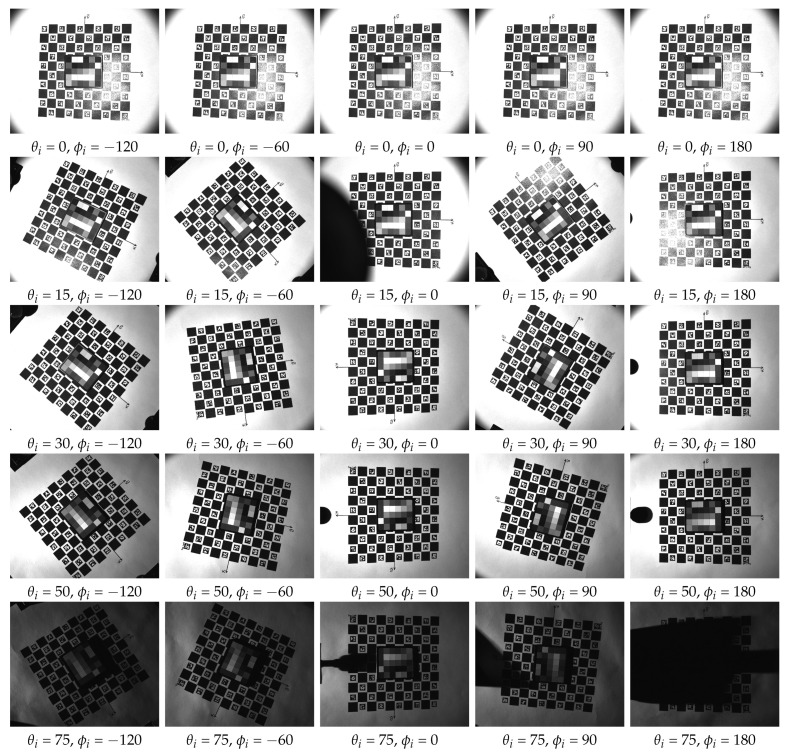
Captured images. The reflection direction is fixed at $\theta_{r}=20$ and $\phi_{r}=0$, while the incident light changes. The images are arranged in rows that correspond to different angles of reflection ($\theta_{i}=0$, 15, 30, 50, and 75 from top to bottom) and in columns that correspond to different directions of reflection ($\phi_{i}=-120$, −60, 0, 90, and 180 from left to right).

**Figure 16:**
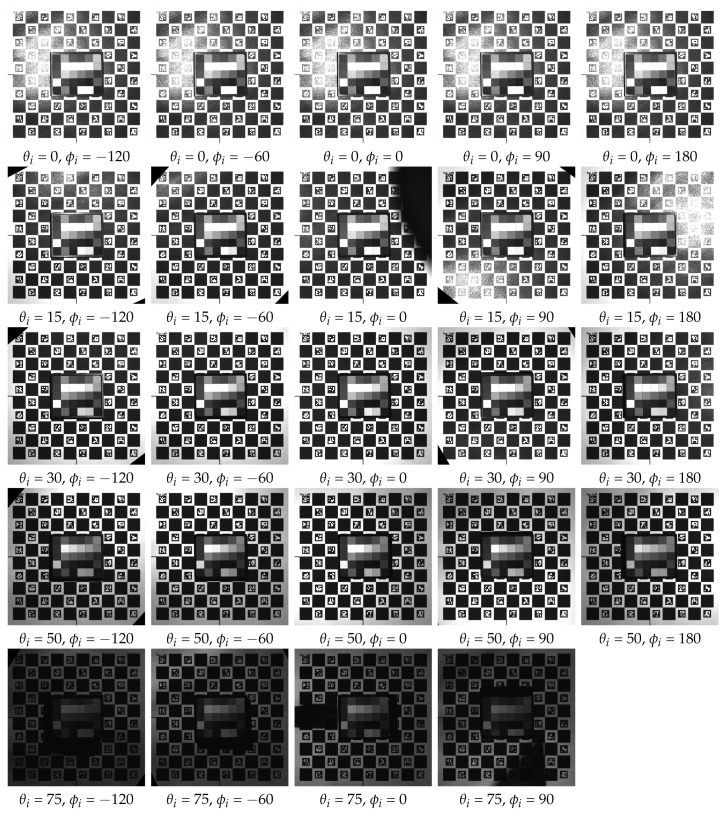
Registered images as output of post-processing stage. The imaging geometries are the same as in Figure 15.

In a second experiment, we maintained the reflection direction at $\theta_{i}=20$ and $\phi_{i}=0$. We captured images of a ColorGauge Micro Target [35] at 25 distinct lighting directions, encompassing directions with zenith angles of $\theta_{r}=0$, 15, 30, 50, and 75, as well as at azimuth angles of $\phi_{r}=-120$, −60, 0, 90, and 180. The raw captured images for the fourth filter and the corresponding post-processed results are illustrated in Figure 15 and Figure 16, respectively. Notably, in the last imaging geometry ($\theta_{i}=75$, $\phi_{i}=180$) as depicted in Figure 15, the shadow caused by the table height adjustment screw obscured the target and ChArUco board, resulting in a failed post-processing for this particular image. Additionally, it is evident from the same figure that as the zenith angles for the illumination direction increase, the images progressively darken. This effect is attributed to the illumination zenith angle; with an increase in this angle, less light is received by the surface area, resulting in darker images.

**Figure 17:**
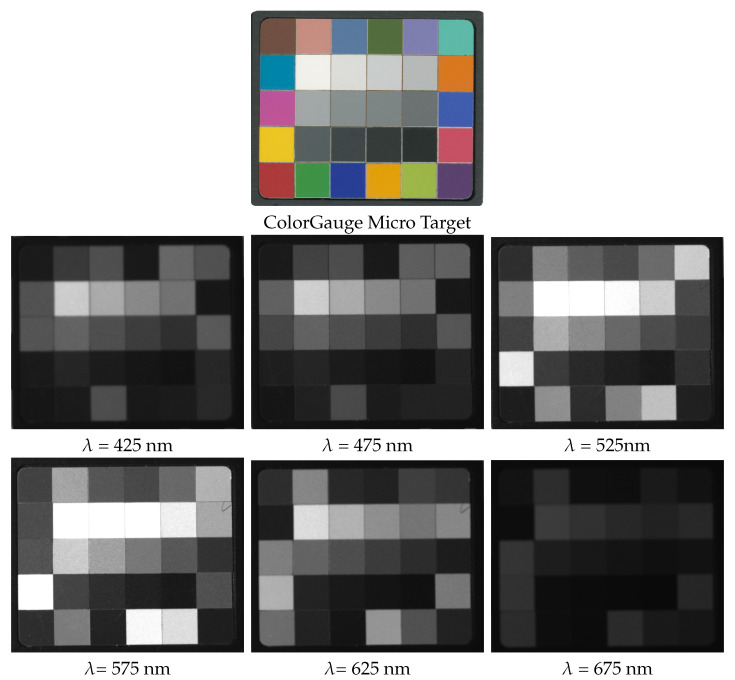
The cropped images of the original color ColorGauge Micro Target [35] and the registered images of different spectral channels for incident direction of ($\theta_{i}=20$, $\phi_{i}=0$) and reflection direction of ($\theta_{r}=0$, $\phi_{r}=-120$).

In the previous figures, to streamline data presentation, we displayed images from a single spectral filter and exposure time. However, the device captures multispectral images with varying exposure times to construct irradiance maps and HDR images. To demonstrate the capability of multispectral imaging, Figure 17 showcases cropped images from different spectral channels captured from the ColorGauge Micro Target, with an incident direction of ($\theta_{i}=20$, $\phi_{i}=0$) and reflection direction of ($\theta_{r}=0$, $\phi_{r}=-120$). The top row presents the color photo of the target, while the second and third rows display corresponding images from different channels. It is worth noting that in the first and last channels, the images appear darker due to lower camera spectral responsivity and reduced power of the light source. Additionally, certain patches with specific colors appear brighter in corresponding spectral channel images; for instance, patches with green colors appear brighter in images from channels with central wavelengths of λ = 525 nm and λ = 575 nm, while they remain completely dark in the blue region (λ = 425 nm).

Furthermore, as evident in these spectral images, different channels exhibit varying levels of sharpness, with central channels being the sharpest. This phenomenon occurs due to chromatic aberration present in the optics of the multispectral camera. Chromatic aberration arises from the physical property wherein the refractive index of objective lenses varies with wavelength. It gives rise to two types of chromatic aberrations: transversal chromatic aberrations, which lead to the displacement of spectral channel images, and longitudinal chromatic aberrations, resulting in differences in focus levels among spectral channel images [36,37].

## 5. Conclusions

In this work, we introduced our innovative multispectral image-based device for measuring SVBRDF and BTF. We outlined our efforts in designing, implementing, and validating this device to bridge existing research and development gaps in the field. Our setup utilizes a rotational table, offering three DFs to rotate the sample, while a fourth DF is achieved through an illumination arm rotating the light source around the sample. Importantly, we considered spectral channel differences in the optical design, a novel aspect not addressed in prior works.

Preliminary measurements have effectively showcased the device’s ability to accurately capture both angular and spectral data. However, we acknowledge limitations, particularly concerning the accessible incident-reflection direction combinations due to physical restrictions. Our future endeavors will be dedicated to overcoming these constraints, refining the device’s functionality, improving mechanical design, and optimizing the measurement procedure. This device stands as a notable advancement in material appearance measurement, promising a deeper understanding of crucial visual properties across various industries.

## Figures and Tables

**Table 1:** The summarized specifications of all three cameras.

Camera	Technology	Spectral Range [nm]	Spectral Channels	Sensor Technology	Resolution	Pixel Pitch [μm]	Bit Depth	Dimension [mm]	Weight [g]
Silios CMS-C	SFA	430–700	8 + 1 *	CMOS	426 × 339	5.3 × 3	10	62 × 62 × 31	110
Silios CMS-S	SFA	650–930	8 + 1 *	CMOS	426 × 339	5.3 × 3	10	62 × 62 × 31	110
SpectroCam VIS + NIR	Filter Wheel	350–1050	8	CCD	2456 × 2058	3.45	12	136 × 124 × 105	680

* 8 color bands + 1 panchromatic.

**Table 2:** Result parameters of the optical design for different spectral channels. For all the filters |p|=46.5 cm, $f^{\prime }=25$ mm, and DoF = 10 cm.

Filter#	λ [nm]	*w* [μm]	*w* [DPI]	pix [μm]	∅D [mm]	F/#
Filter 1	425	226	112	6.43	2.1	11.73
Filter 2	475	239	106	6.80	2.3	11.10
Filter 3	525	251	100	7.14	2.4	10.56
Filter 4	575	263	96	7.48	2.5	10.09
Filter 5	625	274	92	7.80	2.6	9.67
Filter 6	675	285	89	8.10	2.7	9.31

**Table 3:** The summarized specifications of Thorlabs mounted LEDs [31].

LED Model	Correlated Color Temperature (CCT) [K]	LED Output Power (Min/Typ.) [mW]	Irradiance (Typ.) [μW/mm^2^]	Max Current [mA]	Forward Voltage (Typ.) [V]	Viewing Angle (Full Angle at Half Max) [°]
MCWHL6	6500 (Cold White)	990/1430	25.0	1200	2.8	120
MNWHL4	4900 (Neutral White)	740/880	7.7	1225	2.9	150
MWUVL1	4000 (Neutral White)	235/338	4.0	125	6.3	120
MWWHL4	3000 (Warm White)	570/640	9.4	1000	3.0	120

**Table 4:** Results of the spectral reconstruction for LED selection.

LED Model	DE94_D65		DE94_A		DE94_F2		RMSE		GFC	
	mean	std	mean	std	mean	std	mean	std	mean	std
MCWHL6	0.2307	0.1649	0.1575	0.1373	0.3227	0.3992	0.0039	0.0037	0.9989	0.0019
MNWHL4	0.2395	0.2272	0.2156	0.2193	0.4063	0.5217	0.0039	0.0037	0.9989	0.0018
MWUVL1	0.6120	0.5093	0.2628	0.2238	0.6082	0.5670	0.0034	0.0028	0.9990	0.0019
MWWHL4	0.2403	0.1927	0.1781	0.1544	0.3645	0.4239	0.0039	0.0037	0.9989	0.0019

## Data Availability

Data available on request from the authors.

## Abbreviations

The following abbreviations are used in this manuscript:

BRDF	Bidirectional Reflectance Distribution Functions
SVBRDF	Spatially Varying Bidirectional Reflectance Distribution Functions
BTF	Bidirectional Texture Functions
SDG	United Nations’ Sustainable Development Goal
CCD	Charge-Coupled Device
CMOS	Complementary Metal-Oxide Semiconductor
LED	Light-Emitting Diode
CCT	Correlated Color Temperature
RGB	Red, Green and Blue
TAC7	Total Appearance Capture material scanner by X-Rite Incorporated
DF	Degree of Freedom
DoF	Depth of Field
PC	Personal Computer
TI	Texas Instruments
USB	Universal Serial Bus
SFA	Spectral Filter Array
FWHM	Full Width at Half Maximum
VIS	Visible
NIR	Near Infrared
HDR	High Dynamic Range
UV	Ultraviolet
IR	Infrared
CoC	Circle of Confusion
DPI	Dots Per Inch
PCB	Printed Circuit Board
EEPROM	Electrically Erasable Programmable Read-Only Memory
OBSW	Optical Barrier SWitch
SPD	Spectral Power Distribution
OETF	Opto-Electronic Transfer Function
CRI	Color Rendering Index
TLCI	Television Lighting Consistency Index
RLSW	Roller Lever micro-SWitch
RMSE	Root Mean Square Error
GFC	Goodness of Fit Coefficient
